# Five-Year Antimicrobial Susceptibility of *Pseudomonas aeruginosa* from a Local Tertiary Hospital in Bacolod City, Philippines

**DOI:** 10.3390/tropicalmed2030028

**Published:** 2017-07-12

**Authors:** Alain C. Juayang, Joseph Peter T. Lim, Ann Francis V. Bonifacio, Alaica Victoria L. Lambot, Sean Maybelle Millan, Vic Zyrus Jeriko N. Sevilla, Julien Kate T. Sy, Paul John Villanueva, Carmina P. Grajales, Christine T. Gallega

**Affiliations:** 1 Clinical Laboratory, Dr. Pablo O. Torre Memorial Hospital, Bacolod City 6100, Philippines; djozip@gmail.com (J.P.T.L.); gallegachristine@yahoo.com (C.T.G.); 2 Medical Technology Program, Colegio San Agustin, Bacolod City 6100, Philippines; annefrancis301996@gmail.com (A.F.V.B.); lalaicavictoria@yahoo.com (A.V.L.L.); sean_maybelle@yahoo.com (S.M.M.); maigeck@yahoo.com (V.Z.J.N.S.); julienkatesy@gmail.com (J.K.T.S.); pauljohnvil95@gmail.com (P.J.V.); cadekid@yahoo.com (C.P.G.)

**Keywords:** antibiotic susceptibility, *Pseudomonas aeruginosa*, local tertiary hospital, Bacolod City

## Abstract

Over five years, a total of 646 *P. aeruginosa* isolates was acquired from different clinical specimens and their resistance to the commonly used anti-pseudomonal antibiotics was determined. The majority of the isolates were from respiratory (60.99%) and urinary sources (23.22%) while the least came from transudates and exudates (2.01%). Most of the samples were acquired from older adults (77.55%), most of whom were admitted (67.03%). Amikacin was found to be the most effective drug with a resistance rate of 7.5%, followed by piperacillin/tazobactam (8.5%) and gentamicin (13.5%). On the other hand, 26.7% of the isolates were resistant to levofloxacin. Almost 100% of the isolates were screened positive for AmpC production, which may suggest inducible resistance against expanded spectrum beta-lactamase. Furthermore, for the last three years, *P. aeruginosa* isolates from this area have been noted to have decreasing resistance only to aztreonam and gentamicin. Also, for five years, a mean MAR index of 0.17 was noted which indicates either proper antibiotic use or most isolates did not come from high-risk areas. Moreover, there was no significant difference in the resistance of *P. aeruginosa* when compared by specimen source (*p* = 0.662), but significant when compared by year band (*p* = 0.02).

## 1. Introduction

*Pseudomonas aeruginosa* is considered the most medically important among the genus *Pseudomonas* that frequently causes opportunistic and hospital-acquired infections such as pneumonia, urinary tract infections, otitis media and wound infections [1,2,3,4]. It is widely distributed in the environment, particularly water, soil and vegetation, but can also be isolated from stool, throat, nose and moist areas of the skin from a fraction of healthy individuals [2]. Additionally, this bacterium is also considered to be resilient because it can readily colonize and invade epithelial surfaces, undermine host defenses, induce systemic toxicity, and is still associated with considerable morbidity and mortality.

Treatment of *P. aeruginosa* infections is complicated owing to its resistance mechanism due to its impermeable outer membrane. Moreover, it is also adept at acquiring resistance to most antibiotics via a variety of mechanisms by the use of selective porins, production of efflux pumps and by possessing a chromosome that contains an inducible beta-lactamase [1]. All of the aforementioned are instrumental to its intrinsic and acquired resistances. Frequently, *P. aeruginosa* is not susceptible to one or more antibiotics. Thus, therapy must be guided by the susceptibility profiles of individual strains. The emergence of resistance that occurs after 3–4 days after the initiation of treatment [5] that leads to treatment failure can sometimes be resolved by administering two types of antibiotics simultaneously for a synergistic effect [1].

For decades, an increase in the proportion and absolute number of bacterial pathogens resistant to multiple antibiotics has been observed and is now considered an emerging global disease that poses major public health problems [6]. There are reports on the disparities in the characteristics of *P. aeruginosa* such as its antibiotic resistance that varies with geographical location, site of infection [7] and hospital environment [8]. It is, therefore, the goal of this study to determine the prevalence of *P. aeruginosa* in a local hospital in Bacolod City, Philippines and to evaluate its susceptibility against certain antibiotics.

## 2. Materials and Methods

### 2.1. Inclusion and Exclusion Criteria

The data utilized in this study were obtained from the records of the laboratory of the said hospital. It included all the isolates from 2011 to 2015. The isolates were from samples of various clinical sites gathered from patients who were diagnosed with infections caused by *P. aeruginosa.* The data were grouped based on the origin of the sample, on the age groups and gender of the patients, and based on the isolate’s antimicrobial susceptibility test results. In cases where more than one *P. aeruginosa* was isolated from a single patient, the sample that is considered to be the primary source of infection is included (i.e. pulmonary excretions rather than blood in cases of pneumonia).

### 2.2. Isolation and Identification

Submitted samples were analyzed using the traditional culture methods [9,10,11]. Urine samples were inoculated onto blood agar and MacConkey agar. Sputum and tracheal aspirates were inoculated onto blood agar, MacConkey agar and chocolate agar. Blood samples were inoculated onto BACTEC Aerobic Plus vial, while cerebrospinal fluid (CSF) and other body fluids were inoculated onto blood agar, MacConkey agar, trypticase soy broth and chocolate agar.

Identification of *P. aeruginosa* was based on routine biochemical methods [9,10] that included the following reactions: Gram-negative bacilli, oxidase positive, unable to ferment sugars on triple sugar iron, motile, do not produce sulfide and indole, citrate positive, urease negative, lysine decarboxylase positive, lysine deaminase negative, able to grow at 42 °C, and pigmented.

### 2.3. Antibiotic Susceptibility Test

In vitro susceptibility testing utilizing amikacin (30 µg), aztreonam (30 µg), ciprofloxacin (5 µg), gentamicin (10 µg), imipenem (10 µg), levofloxacin (5 µg), meropenem (10 µg), piperacillin/tazobactam (100/10 µg) and tobramycin (10 µg) was determined using the disc diffusion method following the guidelines stipulated in the M02-A11 of the Clinical Laboratory Standards Institute (CLSI) [12]. Results of disc diffusion tests were then interpreted using the M100-S25 [13] document.

### 2.4. AmpC Screening Test Using Cefoxitin

AmpC production was screened using cefoxitin (30 µg) disc in the same manner as performing the in vitro susceptibility testing [14,15]. A zone of inhibition, which is equal to or lower than 18 mm, is considered to be suggestive for an AmpC-producing isolate. AmpC-producing organisms are able to degrade penicillins, expanded-spectrum cephalosporins [14], oxyimino-β-lactams and beta-lactam inhibitors [15].

### 2.5. Multiple Antibiotic Resistance Index

Multiple antibiotic resistance (MAR) index is calculated as the ratio of the number of antibiotics an isolate was resistant to, over the total number of antibiotics it was tested against [16,17]. MAR is commonly used as a tool for health risk assessment to identify whether isolates are from regions of low or high antibiotic use. A MAR index of more than 0.2 indicates that the organism have originated from an environment where antibiotics are frequently used [16,17].

### 2.6. Quality Control

*P. aeruginosa*, with American type culture collection (ATCC) number 27853, was used as the reference strain for quality control of culture media, biochemical tests and susceptibility testing.

### 2.7. Ethical Consideration

The study was approved by the Research Ethics and Review Committee of Dr. Pablo O. Torre Memorial Hospital with approval number DPOTMH RERC 2016-16.

### 2.8. Statistical Analysis

Data analysis was done using the WHONET software 5.6 version downloaded from the World Health Organization website. The age groups of pediatric and adult patients were automatically classified by the WHONET software. A Kruskal-Wallis test was also noted to determine differences as to specimen source and by year band.

## 3. Results

A total of 646 isolates of *P. aeruginosa* was isolated for five years and identified by standard microbiological procedures. Out of these isolates, 25 were isolated from blood, 150 from urine, 64 from wounds and abscesses, 13 from transudates and exudates, and 394 from sputum and tracheal aspirates. When grouped according age, 38 specimens from paediatrics (18 years old and below) and 608 specimens from adults, specifically 32 from young adults (19–35 years old), 75 from mid-adults (36–55 years old) and 501 from older adults (56 years old and above). As to the location of these patients, 181 came from the outpatient department, 32 from intensive care units and 433 from inpatients (non-ICU).

Generally, out of the eight antibiotics tested, amikacin had the least resistance of 7.5%, followed by piperacillin/tazobactam with 8.5% and carbapenems, imipenem and meropenem, with 16% and 17.1% respectively. Highest resistance was observed against levofloxacin with 26.7%.

**Figure 1:**
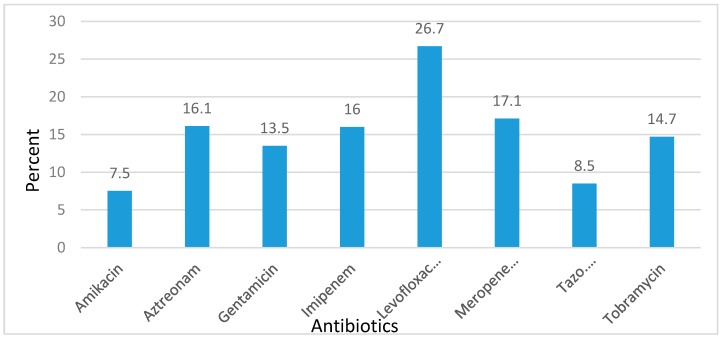
Five-year antimicrobial resistance of *P. aeruginosa* to selected antibiotics.

Figure 1 shows the antimicrobial susceptibility profile of *P. aeruginosa* over the five-year period, while Table 1 indicates the percentage resistance of *P. aeruginosa* against certain antibiotics per year.

As for the five-year trend, Table 2 summarizes the resistance of *P. aeruginosa* to selected antibiotics per year. As shown in Table 1, most of the antibiotics do not have a consistent trend. Noting that irregular resistance is observed except in some antibiotics such as aztreonam and gentamicin where a decrease in resistance was observed in the last three years.

Specimen-wise, the resistance patterns of *P. aeruginosa* to the antibiotics are almost identical. Amikacin and piperacillin/tazobactam are still the most effective as compared to other antibiotics, and levofloxacin was still noted to have the highest resistance. Additionally, as noted in Table 2, resistance to cefoxitin may be suggestive of AmpC production indicating resistance to penicillins, beta-lactam inhibitors and third generation cephalosporins [18].

The five-year MAR index of the isolates is shown in Table 3. As noted, the majority of the isolates (75.2%) fall under the MAR index of 0.2, as the mean MAR is only 0.17. This indicates that the majority of the isolates were not from regions or areas with high risk source of contamination [16,17].

Lastly, using a Kruskal-Wallis test, it revealed that there was no significant difference in the resistance of isolates when compared between specimen sources (*p* = 0.662). However, when the resistances were compared as to the year band, significant difference is noted (*p* = 0.02).

## 4. Discussion

*P. aeruginosa* is commonly isolated in the environment and as such it is termed as ubiquitous. It is present in soil, water, plants, humans, animals and even in healthcare facilities [19]. These serve as major reservoirs that may lead to nosocomial infections [20]. It has the ability to colonize healthy individuals and acts opportunistically. It is deemed harmful in hospitals and poses quite a significant threat [19]. Additionally, this bacterium has the ability to adapt to various environmental challenges [21] and tends to be difficult to treat owing to the presence of its R factor that carries the gene that determines its resistance to antibiotics [20]. This makes *P. aeruginosa* one of the more notorious pathogens of nosocomial infection.

Identification of the isolate as *P. aeruginosa* is already an indication that the isolate is an AmpC β-lactamase producer [15]. That is, the organism has the potential to readily mutate and develop resistance during therapy to β-lactam drugs, except for carbapenems and zwiterionic cephalosporins [22]. Mortality rates for *P. aeruginosa* infections range from 18% to 61% even with successive advances in antimicrobial therapy [21]. This makes *P. aeruginosa*-associated infections alarming.

As with this study, *P. aeruginosa* infection was primarily noted among older adults (*n* = 501, 77.5%) particularly causing pneumonia (*n* = 394, 60.9%). There are a number of reasons why older adults are burdened by this type of infection. These include age-associated impairments in immunity that lead to reduced response to vaccination, a constellation of chronic and comorbid diseases, and functional limitations associated with advanced age [23,24]. Additionally, older adults are at risk for aspiration pneumonia, outbreaks of gastroenteritis, recurrent urinary tract infection, and prosthetic device infections [20].

As observed in this study, most tested antibiotics, except levofloxacin, can still be used as a form of empiric therapy, noting that they have a resistance rate lower than 20%. *P. aeruginosa* isolates were noted to be most resistant to the fluoroquinolone levofloxacin, with a resistance rate of 26.7%. It is also important to note that the isolates were most susceptible to amikacin (7.5%), piperacillin/tazobactam (8.5%), and gentamicin (13.5%). For the reason that the isolates were not tested molecularly for gene similarities, the resistance pattern observed in this study could be due to clonal spread of *P. aeruginosa*.

According to Berglund [25], one of the reasons for resistance among bacteria is a result of either overuse and misuse of antibiotics. By misuse, this refers to the prescription of antibiotics without establishing bacterial infection, and the non-compliance of the patient to the full prescription. Moreover, antibiotic resistance can also be transferred horizontally between bacteria.

The resistance rate of *P. aeruginosa* in this tertiary hospital in Bacolod City revealed that it is almost similar to the countrywide data of ARSP [26] and to other researchers in other Asian nations. This current study reveals a meropenem resistance of 17.5% which is slightly higher compared to that of the ARSP [26] with only 15.5%, lower compared to that found in Malaysia [27] with 21% and Iran [19] with 58.5%. The resistance rate to piperacillin/tazobactam of this study (8.5%) is also comparable to that of Pathmanathan [27] in Malaysia with 7% and lower than that of the data of ARSP [26], which is 17.1%. Amikacin had the lowest resistance rate in this study, which is also on par with the data of ARSP [26], as well as with the study of Pathmanathan [27] in Malaysia, and Sedighi [19] in Iran.

Amikacin in this study is noted to be the most effective drug. However, because of its numerous side effects including renal toxicity, blurred vision, hearing loss, Bartter-like syndromes [28], neuromuscular blockade, arthralgia, apnoea and many more, it is not commonly used. Thus, using the data of this study, the use of piperacillin/tazobactam will be a good choice for empiric therapy. Among the different classes of antibiotics, this study shows that *P. aeruginosa* has lower resistance to aminoglycosides amikacin, gentamicin and tobramicin. This is because aminoglycosides are multifunctional hydrophilic sugars that possess several amino and hydroxyl functionalities that are able to inhibit prokaryotic protein synthesis [29]. This is also seen in the study of Pathmanathan [27], Sedighi [19], and Micek [30].

Resistance to carbapenems that include imipenem (16%) and meropenem (17.1) was also noted in this study. This is quite alarming, taking into account that carbapenems are the last line of antibiotics for treating Gram-negative bacilli infections. Resistance to carbapenems may be due to a result of complex interactions of several mechanisms including production of carbapenemase, overproduction of efflux system and loss of outer membrane porins. *P. aeruginosa* isolates that are carbapenem resistant, specifically carbapenemase producing, are the worst, for the reason that they are associated with a higher mortality rate [21].

Significant difference as to year band is noted wherein the resistance of the bacterium had gone beyond the 20% mark. During these years, it is advisable that these antibiotics are not to be used for empiric therapy. However, if the resistance rate is compared to specimen source, it can be noted that the antibiotics that are effective on one anatomical source is almost identical in terms of efficacy when used on other sources.

Having a mean MAR index of 0.17, this study suggests that most of the isolates were from an area or region where antibiotics against *P. aeruginosa* are not abused nor taken for granted. In other words, the control measures in monitoring the spread of infections are in place in the area of study and are religiously practiced. However, even though the majority of the isolates (75.23%) had a MAR index of below 0.2, a number of isolates (24.77%) had a MAR index of more than 0.2, and 15 of which had a MAR index of 1.0. This indicates that the patients where these isolates came from were exposed to an environment of high-risk contamination from a region or area where there is high antibiotic use [16,17].

Overall, the data obtained in this study indicate that conventional drugs being used for *P. aeruginosa* are still effective provided that they are below the 20% cut-off mark, except for levofloxacin. Though effective, monitoring the antibiotic resistance of the said pathogen is necessary, noting that it is one of the bacteria capable of having accelerated resistance to most antibiotics. This study also stresses the importance of microbiological analysis provided by the microbiology laboratory so that clinicians can select the appropriate antibiotic therapy for timely treatment.

## 5. Conclusions

*P. aeruginosa*-related infections are still prevalent among patients and are commonly affecting older adults. The resistance rate of the bacterium for five years from this tertiary hospital indicates that it can still be treated by any anti-pseudomonal drug mentioned in this study and that antibiotic policies exist and are being carried out. However, certain observed resistance, especially to carbapenems, was concerning. These isolates may act as sources of infection that threaten the health of patients and communities. Thus, prudent use of antibiotics is still recommended to limit further increase of resistance.

## Figures and Tables

**Table 1:** Percentage resistance of *P. aeruginosa* to certain antibiotics from 2011 to 2015.

Antibiotics	2011 (*n* = 102)	2012 (*n* = 68)	2013 (*n* = 184)	2014 (*n* = 142)	2015 (*n* = 150)
Amikacin	8	12	11	4	5
Aztreonam	16	15	22	18	9
Gentamicin	20	12	16	11	9
Imipenem	13	10	18	29	8
Levofloxacin	36	29	23	44	9
Meropenem	18	27	21	21	8
Piperacillin/tazobactam	5	8	8	11	8
Tobramycin	19	10	16	20	8

**Table 2:** Five-year percentage antibiotic resistance of *P. aeruginosa* with respect to specimen source.

	Respiratory (*n* = 394)	Urinary (*n* = 150)	Blood and CSF (*n* = 25)	Wounds and Abscess (*n* = 64)	Transudates and Exudates (*n* = 13)
Amikacin	6.7	6.7	0	8.5	0
Aztreonam	17.8	12.9	6.2	8.7	53.8
Cefoxitin	96	95.1	100	100	100
Gentamicin	13.8	15.8	28.6	9.3	0
Imipenem	15.1	11.5	18.8	6.1	53.8
Levofloxacin	25.7	3.6	6.7	12.1	0
Meropenem	19.3	13.5	5.9	4.4	53.8
Piperacillin/tazobactam	8.3	9	0	2.4	53.8
Tobramycin	12.1	18	21.4	7.5	0

**Table 3:** Multiple antibiotic resistance of *P. aeruginosa* in five years.

MAR Index	Frequency Among Isolates
0	271
0.1	136
0.2	79
0.3	41
0.4	32
0.5	29
0.6	28
0.7	8
0.8	3
0.9	4
1.0	15
